# Personogenesis Through Imitating Human Behavior in a Humanoid Robot “Alter3”

**DOI:** 10.3389/frobt.2020.532375

**Published:** 2021-01-18

**Authors:** Atsushi Masumori, Norihiro Maruyama, Takashi Ikegami

**Affiliations:** ^1^Department of General Systems Science, University of Tokyo, Tokyo, Japan; ^2^Alternative Machine Inc., Tokyo, Japan

**Keywords:** personogenesis, agency, imitation, self-simulation, memory, reconsolidation, humanoid robot

## Abstract

In this study, we report the investigations conducted on the mimetic behavior of a new humanoid robot called Alter3. Alter3 autonomously imitates the motions of a person in front of it and stores the motion sequences in its memory. Alter3 also uses a self-simulator to simulate its own motions before executing them and generates a self-image. If the visual perception (of a person's motion being imitated) and the imitating self-image differ significantly, Alter3 retrieves a motion sequence closer to the target motion from its memory and executes it. We investigate how this mimetic behavior develops interacting with human, by analyzing memory dynamics and information flow between Alter3 and a interacting person. One important observation from this study is that when Alter3 fails to imitate a person's motion, the person tend to imitate Alter3 instead. This tendency is quantified by the alternation of the direction of information flow. This spontaneous role-switching behavior between a human and Alter3 is a way to initiate personality formation (i.e., personogenesis) in Alter3.

## 1. Introduction

We present a new humanoid robot named Alter3 (**Figure 2**) and analyze the dynamics of Alter3's interactions with humans. The philosophy behind Alter3 is grounded in long-running discussions around human/robot cognition (see section 2). We are particularly interested in Rössler's argument of an artificial cognitive map system (Rössler, 1981), and we attempt to realize and extend his ideas with Alter3. Rössler named the self-organization of a dynamic cognitive map under locomotion as the “Helmholtz–Poincare–Tolman” hypothesis based on Helmholtz's internal map system generated through locomotion (Von Helmholtz, 1867), Poincare's internal and external representation of the world (Poincarẽ, 1905), and Tolman, O'Keefe, and Nadal's ideas of a cognitive map, which was later discussed in relation to placing cells in the hippocampus (O'Keefe and Nadel, 1978).

Dayan et al. (1995) later argued that Helmholtz's idea could be implemented in a self-supervised hierarchical neural system, which they called a Helmholtz machine. The Helmholtz machine is based on an inference system that uses variational Bayesian networks. It is essentially equivalent to a Boltzmann machine (Hinton and Sejnowski, 1983) and provides a basis for a variational autoencoder (Kingma and Welling, 2014).

Apart from the probabilistic approach to cognitive map systems, a dynamic systems approach has also been studied. Jun Tani, for example, studied the self-organization of a neural representation of an environment, with a recurrent neural network on a navigation robot in a real environment (Tani, 1996). More recently, using long short-term memory networks, Noguchi et al. (2019) demonstrated the modality of self-organization of a cognitive map in a navigation robot. The current research is not a probabilistic approach to cognitive map systems. However, it is not, in the strict sense, a dynamic systems approach, as the updates of the entire system are not synchronized, and above all, it can only operate as a system when it interacts with humans.

Rössler's autonomous navigation system is based on a digital scanner and a digital flight simulator. Alter3 is the realization of another autonomous machine, with a completely new purpose. The purpose is to investigate the ways in which a humanoid robot becomes a person, which we call the “personogenesis” (Rossler et al., 2019) of a humanoid robot. “Personogenesis” refers to the process by which an agent acquires free will to act out of its own volition, much like an independent person. In addition, it may perceive happiness from the emotions of a person or be able to display similar emotions. For example, human babies imitate the mother's facial expressions automatically, which is called primitive mimicry (Meltzoff and Moore, 1989), and then advance to the personogenesis phase. In Rossler et al. (2019) and Rossler (1987), this advancement is initiated by two coupled agents: “the two mirror-competent brain equation carriers with cognition and memory and mirror competence suddenly become, if coupled in a cross-caring fashion, their own masters.” In other words, coupled agents (one of the two can be a real person) can suddenly share and exchange happy mental states with each other. Our primary goal is to observe the transition from the primitive automatic mimicry phase to personogenesis in a humanoid robot.

Alter3 autonomously imitates the motion of a person in front of it and stores those motions in its memory in the form of a time series. At the same time, the self-simulator included in Alter3 simulates Alter3's motions and generates a self-image. If the visual perception (the motion of the person being imitated) and the self-image differ significantly, Alter3 retrieves a motion from memory that is closer to the human motion and enacts the retrieved motion. In both the cases, Alter3's spontaneous neural dynamics affect the generation of motion. Thus, Alter3 involves three primary functions/features: an automatic mimicry capacity, self-simulation, and memory selection/variation with a neural noise source. To the best of our knowledge, this is one of the first study to focus on memory-driven imitation in a humanoid robot.

### 1.1. Automatic Mimicry Capacity

Piaget's major assumption in his cognitive development theory (Piaget, 1966) is based on mimicry. It is known that newborn infants automatically imitate the facial and manual gestures of adults (Meltzoff and Moore, 1989). This ability is believed to be an innate characteristic and is observed in human babies when they are approximately 3 months old. In the design of Alter3, imitation is considered an important step in the development of cognitive abilities. Therefore, we implemented an algorithm that imitates the motion of a person captured by the eye camera.

### 1.2. Self-Simulation

A self-simulator forms a mental image of the self. Recently, David Ha and Jürgen Schmidhuber worked on model-based reinforcement learning and proposed a “world model” (Ha and Schmidhuber, 2018). In this model, an agent learns an environmental model that includes its behavior and uses the environmental model for simulation. It demonstrates that a control policy can be trained in the simulated world.

While these are examples of self-simulators that include not only the self but also the environment, Alter3's self-simulators are more specific to the self-image. A more pertinent study is that of the self-modeling agent proposed by Bongard et al. (2006). Because a four-legged agent acquires a self-model by autonomously generating its own behavior, even if one of the legs is removed, the self-model is able to adapt. Kwiatkowski and Lipson (2019) extended this study by replacing the self-model with a neural network.

In these studies, the self-simulator is autonomously acquired through evolutionary processes or through learning by neural networks; however, in our study, we assume that the self-simulator has already been acquired in Alter3, and the parameters are fixed. This is done to focus specifically on the acquisition of individuality, based on the development of memory through the imitation of human motion.

### 1.3. Memory Selection and Variation

As soon as Alter3 generates a motion, it stores the motion pattern in its memory buffer. The memory is realized as a queue of chunks (3 s each), with a size of 50 chunks (= 1,500 frames). When the memory is full, the oldest memory chunks are removed, and new memory chunks are added to the queue (i.e., first in, first out).

Alter3 imitates the behavior of the person in front of it (this is called the awake or open-eye mode). Alter3 uses the memory queue when it is difficult to imitate behavior or when no human is in front of it. It searches for the optimal behavioral pattern evaluated by the optical flow in the memory chunk. When a memory is retrieved and executed, it is modified by the neural state. This allows the memory to be recalled and rewritten without the presence of a person. Specifically, after the recalled motion is executed, it is combined in spontaneous neural activity to be stored as a slightly different motion. The more it is recalled, the more the memory makes a slightly deformed copy of itself. It can be seen as a Darwinian evolutionary process of the memory. This is called the dream mode or the closed-eye mode.

The details of these algorithms are given in section 3.

## 2. Related Works on Imitation in Humanoids Robots

Imitation of human behavior by humanoid robots is a long-standing theme in terms of cognitive and biological aspects (see e.g., Schaal, 1999). There are two types of imitation studies in robotics: one for learning and the other for communication. Both share the same underlying mechanism of imitation, while the former uses imitation as a learning tool with an explicit purpose, the latter has no specific purpose for imitation besides communication.

Schaal (1999) claimed that imitation would be a promising approach for developing cognition in a humanoid robot. In the recently surveyed article by Hussein et al. (2017), learning through imitation is presented as a viable research area for novel learning methods. Although most works on imitation consider it as a strategy for learning from humans unidirectionally, we are more interested in bidirectional imitation learning—human to robot and robot to human. We call this approach “imitation for communication.”

Through communication, people develop the social ability to think about others and maintain a good relationship, and imitation plays a significant role in this process. As in Trevarthen's experiments (Trevarthen, 1977) with infant–mother communication, and Nadal's study on pretend-play behavior between two children, imitation is a strong driving force for organizing lively interactions (Nadel et al., 2004). Christopher Nehaniv and Kerstin Dautenhahn edited a book on imitation and social learning (Nehaniv and Dautenhahn, 2007). They also started the Aurora project, which aims to help autistic kids acquire social skills with the use of robots (The AuRoRA Project, 1998).

Along with the “imitation for communication” approach, Ikegami and Iizuka (2007) and Iizuka and Ikegami (2004) studied a turn-taking game to show how imitation emerges as a by-product of mutual cooperation. The present work is a continuation of the previous approaches, in a new humanoid body, with new memory dynamics and a self-simulator.

## 3. System Architecture

**Figure 1:**
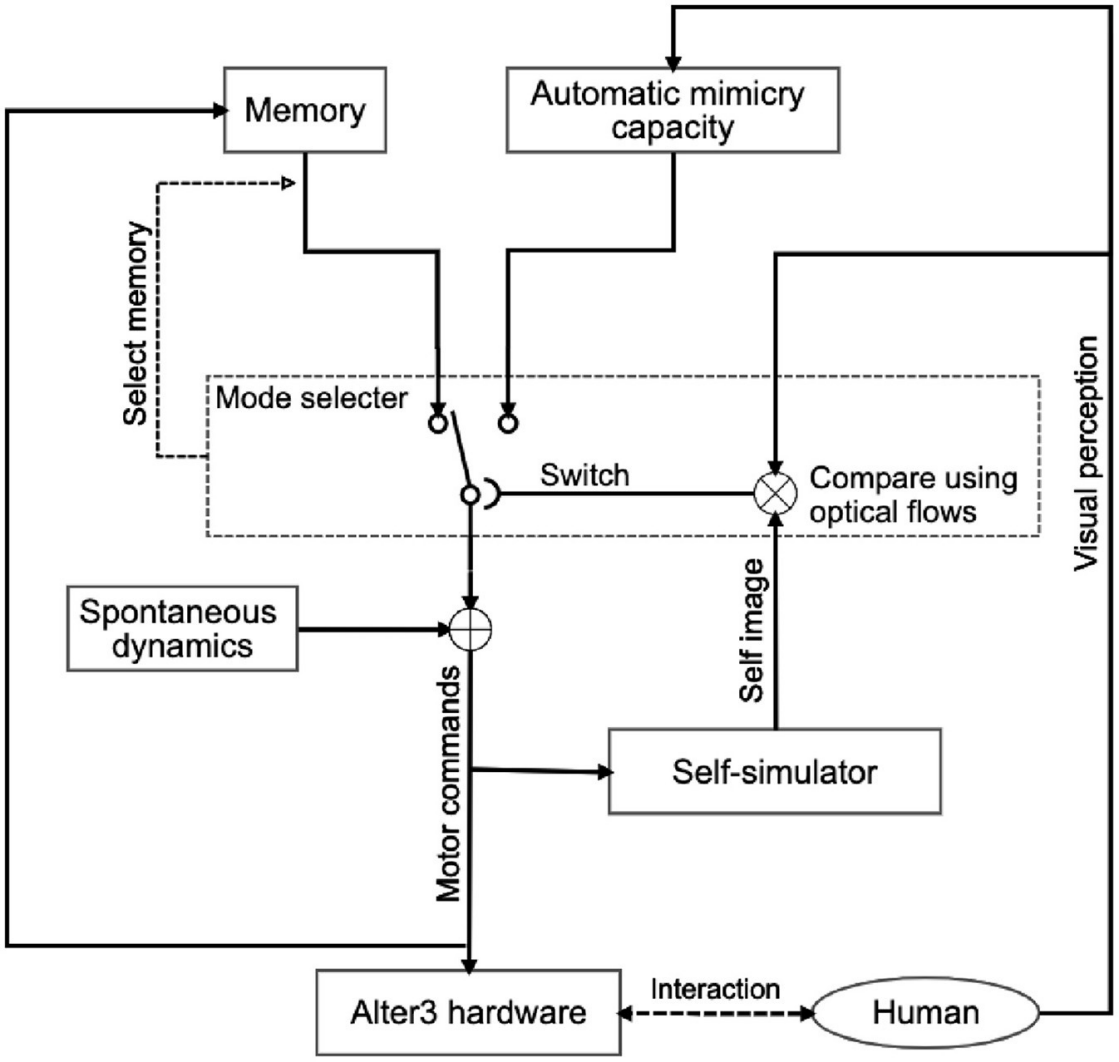
System architecture of Alter3 for the imitation of human behavior. Alter3's motion is controlled by three main subsystems: a self-simulator, an automatic mimicry unit, and memory storage. Additionally, autonomous neural dynamics perturb the memory system. When Alter3 retrieves a memory chunk and executes it, the retrieved chunk is varied with the neural states and stored again. The details of each module are described in section 3. The mode-selection mechanism is also described in the section and Figure 5.

Figure 1 shows an overview of Alter3's internal system. The system is a combination of Rössler's autonomous cognitive map system (Rössler, 1981) and Frith, Blakemoore, and Wolpert's comparator model (Frith et al., 2000). We extended it to include a memory state and a neural network as a spontaneous dynamics circuit. As mentioned earlier, the system is constructed with three functionalities in mind:

1. Automatic imitation capability.
2. Self-simulation.
3. Memory selection and variation through spontaneous dynamics.

In this section, we explain the methods used to achieve the above three functionalities and describe Alter3's hardware.

### 3.1. Humanoid Alter3

**Figure 2:**
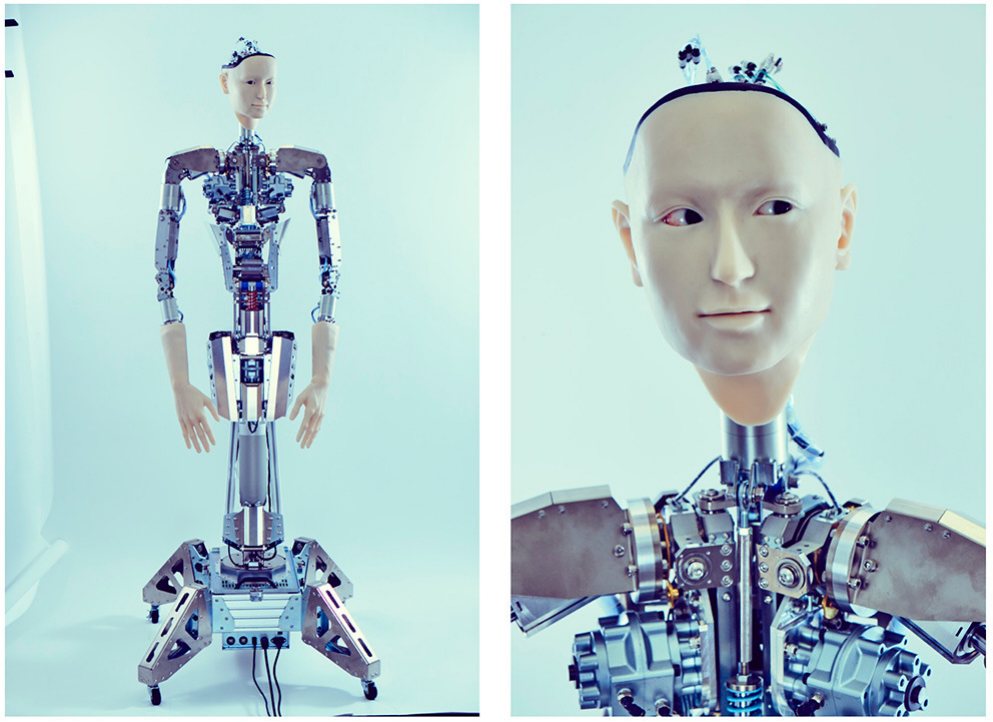
Body of Alter3. The body has 43 axes that are controlled by air actuators. It is equipped with a camera inside each eye.

Alter3's body has 43 movable air actuator axes, and its motions can be controlled through a remotely placed air compressor that is mediated by a control system (Figure 2). More specifically, its motion is controlled by two types of commands: SETAXIS and GETAXIS. A SETAXIS command, which can be regarded as a motor command, is used to set each axis of the humanoid robot to a desired value. By contrast, a GETAXIS command is a command used to retrieve the current axis angle realized on Alter3. Ideally, it is expected that the value obtained from GETAXIS will be the same as the value set by SETAXIS. However, the actual value set for each axis can differ from the intended value. Such differences are caused by physical constraints and latency owing to the body being driven by air actuators. The control system sends commands via a serial port to control the body. Alter3's motions are determined online, and the refresh rate is 100–150 ms.

Alter3 has two cameras, one in each eye, which send visual images to a control system. The camera images are used to extract the key points of the skeleton posture of a human in front of Alter3, using a software called OpenPose (Cao et al., 2017). Alter3 uses the key points of the skeleton to imitate the human posture. In the following sections, the image processing system used for imitation is described in detail.

#### 3.1.1. Automatic Mimicry Capacity

In the awake mode, Alter3's motor commands are generated by the automatic mimicry module through the following processes:

1. Detect a human pose.
2. Map the detected human pose to the angles of the axes.
3. Generate motor commands from the obtained angles and Alter3's spontaneous neural dynamics.

An image from the eye camera is taken as input to a pose detection algorithm. We used OpenPose (Cao et al., 2017) as the algorithm. It detects human poses and generates the positions of key points, such as the head, neck, shoulders, elbows, and wrists. The configuration of the key points of a human skeleton differs from that of the axes in Alter3, and angles of the axes are required as motor commands for Alter3; therefore, we map the positions to the angles. The components responsible for these processes partially constitute Alter3's body schema and can be regarded as the controller in the comparator model (Frith et al., 2000). When OpenPose detects poses of multiple people, Alter3 focuses on the center-most person in its visual field and imitates the person's pose. Once the person is locked into Alter3's vision, the person is tracked until the person disappears from its view.

Alter3's spontaneous dynamics consist of spiking neurons (Izhikevich, 2003) that are combined with the calculated angles of the axes as a weighted average to calculate the final axis values (see details in the following sections). The final values are sent to Alter3 as motor commands at every frame, and Alter3 behaves in accordance with the motor commands. Thus, Alter3 not only imitates human motion but also modifies its own motion to an extent based on its spontaneous dynamics.

It should be noted that the choice of whether Alter3 imitates human motion based on the above-mentioned process (awake mode) or based on its memory (dream mode) depends on the result of the comparison between its self-simulation and current visual perception, as described below.

#### 3.1.2. Self-Simulation

**Figure 3:**
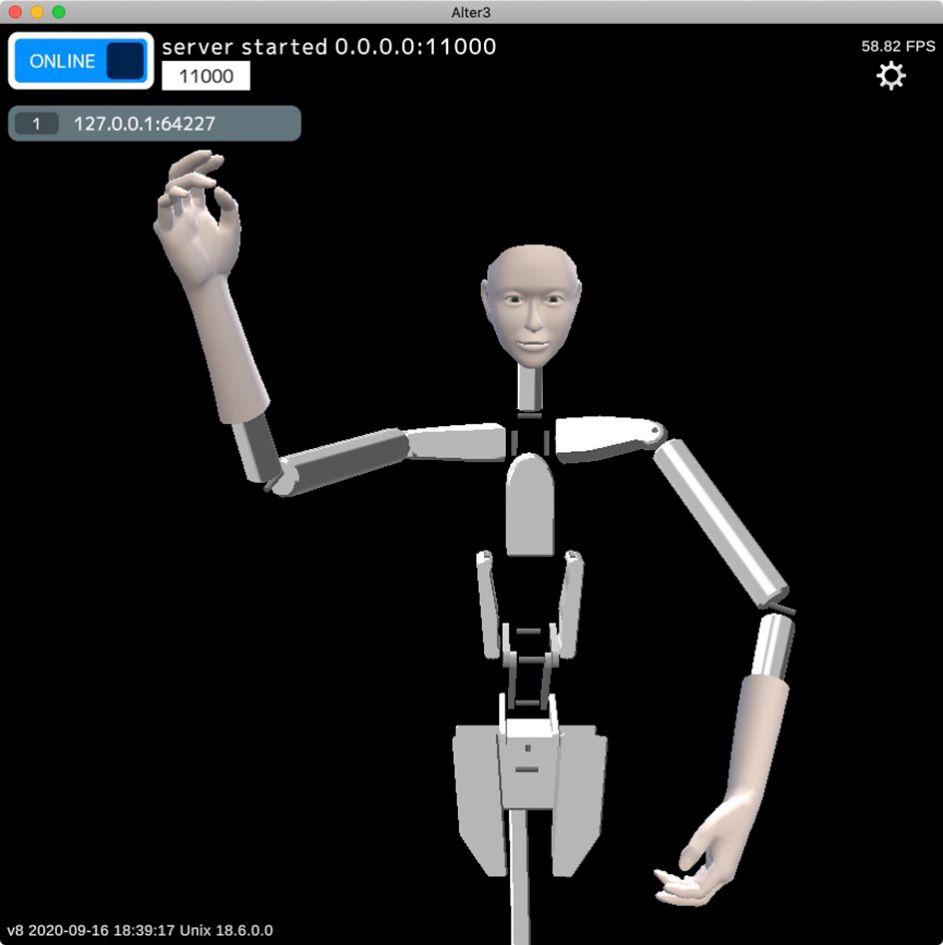
Example of an internal image generated by the self-simulator. The self-simulator receives the SETAXIS commands (motor commands) and generates a visual image.

Alter3 contains a self-simulator that simulates a future self-image before executing motor commands. The self-simulator is a robot simulator that receives each joint angle as a motor command (which is the same as the SETAXIS command described above) and returns a posture as a visual image (Figure 3). We used a custom-built simulator that visualizes the results of forward kinematics by calculating joint positions from joint angles without a physics engine, other than simple inertia. As Alter3's axes are controlled by air actuators that do not have sufficient torque to control the axes precisely, the actual motions differ from the motor commands. Thus, we manually calibrated the upper and lower limits of the joint angles in the simulation by comparing the simulated poses and the actual poses by Alter3. This self-simulator can be regarded as a predictor in the comparator model (Frith et al., 2000), which predicts a future state from an efference copy.

The predicted future self-image is compared with the visual perception of the optical flow values. The difference between the two is used to determine the operation mode of Alter3. If the difference between the optical flow values and the predicted self-image exceeds a threshold, the mode switches from awake mode to dream mode, i.e., Alter3 will stop using its automatic mimicry capacity (OpenPose and its mapping function) and will begin using its memory to generate new imitation behavior. The details of this process are explained in the following subsection.

Therefore, Alter3 uses the self-simulator to predict a future posture from the motor commands generated by the automatic mimicry module before executing the commands. It then determines whether it should execute these commands or use memory to imitate the human motion (based on a comparison between the state predicted by the self-simulator and a target human motion).

#### 3.1.3. Memory Selection and Development

Alter3 has a fixed memory size in which the sequence of movements is divided into short chunks that are stored over time. Each memory chunk is a short sequence of behavior but is labeled by an abstract representation of the visual image of the movement. Specifically, we used the optical flow of the self-image for this purpose. When Alter3 identifies that the automatic imitation of a human is not viable under certain criteria, it searches for the optimal movement in its memory by using the labels. In addition, the movement that is retrieved is stored in the memory as a new memory chunk, which allows the formation of a closed loop.

**Figure 4:**
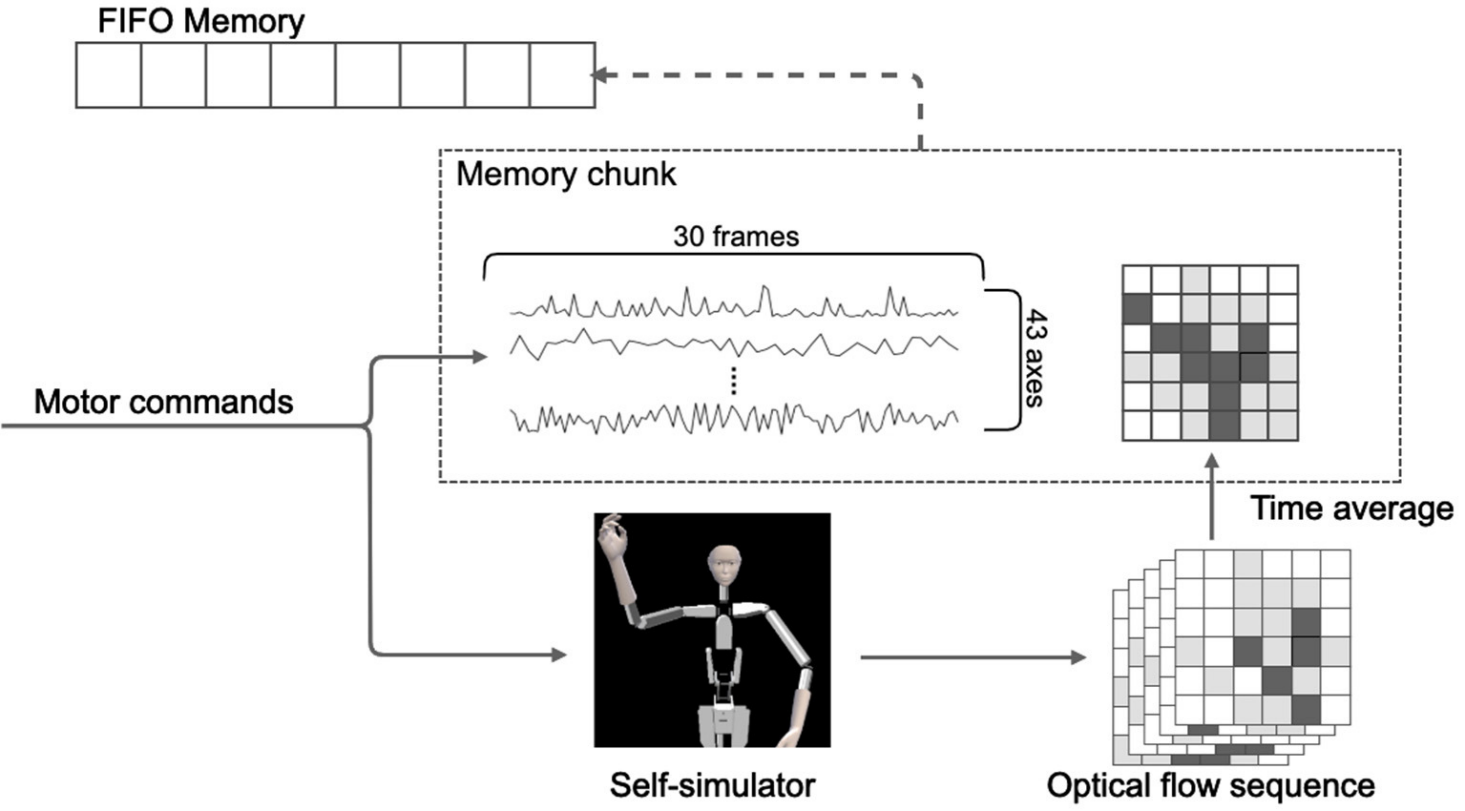
Illustration of the structure and storage process of the memory. The memory comprises a time sequence of action chunks of 30 frames each and a time-averaged value of optical flow associated with each action chunk.

Alter3 stores the executed motor commands in its memory as a memory chunk for every 30 frames. As mentioned in the subsection above, the sequence of motor commands is converted to a self-image via the self-simulator. They are then converted to a series of optical flows. We adapted a dense (lattice) type algorithm to calculate the optical flow. It was originally a two-dimensional vector field, but we adapted it as a scalar field by using the magnitude of the vector. The memory chunk containing 30 frames of the pose sequence was labeled with the time average of the optical flow. Here, we considered the time average of the optical flow as the short-term meaning or label of appearance of the self-motions. For example, when Alter3 performs the action “raising left hand,” the motor command is a high-dimensional time series and contains a large amount of information that is irrelevant to the meaning of the motion. It is assumed that the spatial pattern of the optical flow will always take a high value near the upper right side of the body in such cases. Thus, optical flow is qualified as the meaning or the label. In our experiment, optical flow was calculated using the algorithm proposed by Farnebäck (2003), and OpenCV library (Bradski, 2000) was used for the actual implementation. The memory was realized as a queue of memory chunks, and its size was limited (50 chunks = 1,500 frames). Thus, if the memory was full, the oldest memory chunk was removed, and a new memory chunk was added (i.e., first in, first out). Figure 4 shows this process.

Alter3 can replay past motions based on memory in the dream mode. This memory recall and motion replay occurs in the following two cases.

1. When no human is in sight.
2. When a self-simulated motion differs significantly from the target human motion.

The first case is specifically defined for when OpenPose detects no humans for 100 frames. In this case, Alter3 recalls a motion sequence randomly from memory and replays it. When replaying the motion, Alter3's spontaneous dynamics, which consist of spiking neurons, causes a minor change in the motion as with the case of automatic mimicry (the details of this mutation process are described in the next subsection). The mutated motion is then stored as a new memory. In this case, the memory is reconstructed by store-replay cycles and spontaneous dynamics, without any inputs from the environment. This is similar to memory consolidation in a dream, where memory is reactivated and reorganized (e.g., Wamsley et al., 2010). When a human comes into sight, Alter3 switches to the awake mode.

**Figure 5:**
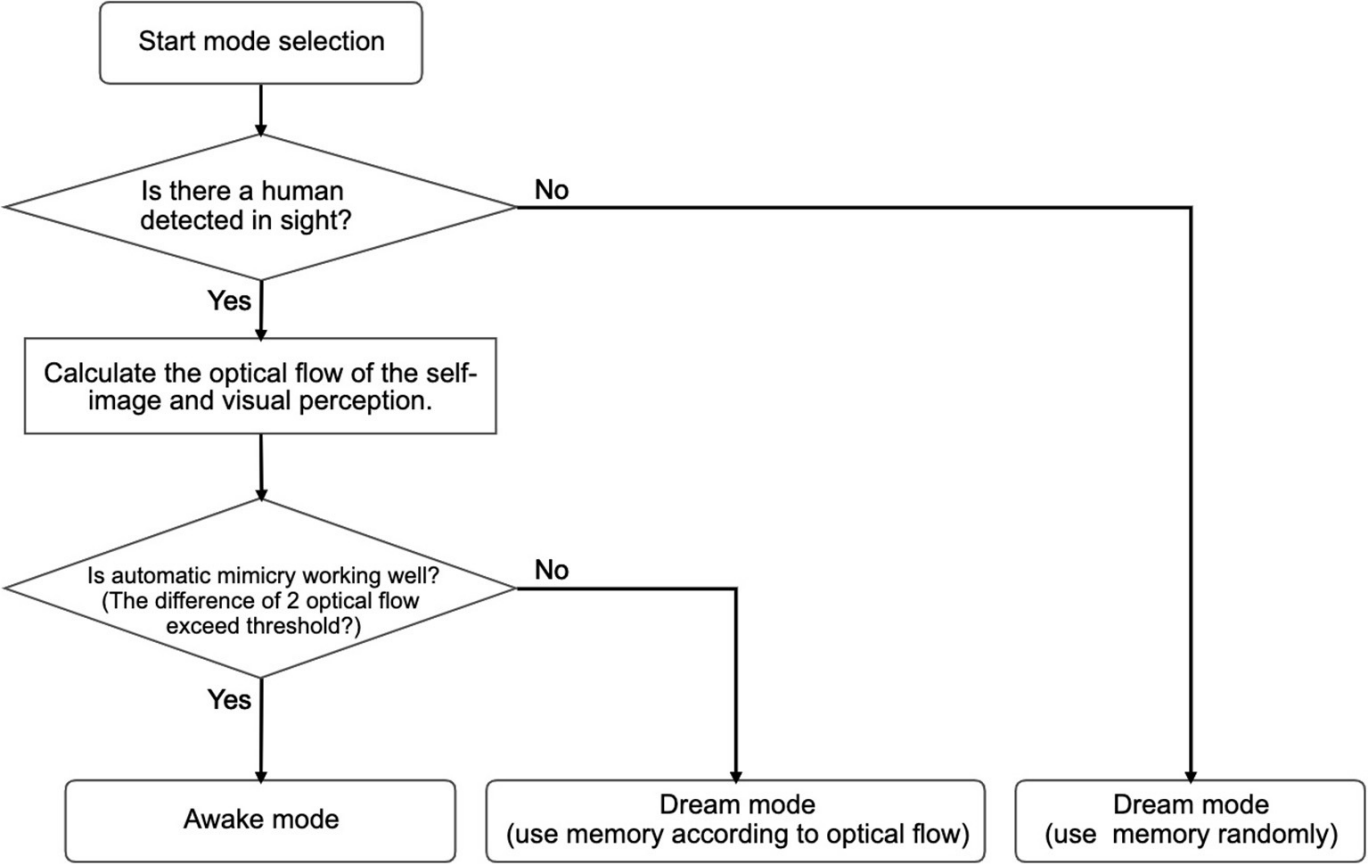
Flow chart for mode selection. When no human is detected and the automatic mimicry module works well, Alter3 enters the awake mode. In the awake mode, Alter3 behaves only according to the automatic mimicry system. A dream mode is divided into two sub-modes. When no person is detected, Alter3 randomly extracts one memory chunk of behavior and replays it. By contrast, when a person is detected, and the automatic mimicry system is unable to imitate the person efficiently (judged by the optical flow), Alter3 searches for a better memory chunk and deploys it.

The second case is specifically defined for when the difference between the optical flows of the self-simulated visual images and the optical flows of the visual perception (human image) exceeds a certain threshold during a short period (15 frames). Mean squared error is used to measure the difference between the two optical flows. In this case, Alter3 retrieves a memory chunk that has been labeled with the optical flow values that are closer to those of the current visual image from the camera and replays the motion. The replayed motion is also mutated by the spontaneous dynamics. The motion is labeled as having a certain optical flow and is stored as a new memory. This is similar to memory reconsolidation, where the recalled memory becomes temporally unstable; then, the memory is consolidated again and becomes stable (e.g., Suzuki et al., 2004). If the optical flow values of the recalled motion are close to the values of the current human motion when a memory chunk is replayed, then Alter3 switches back to awake mode. The algorithms for mode selection are summarized in Figure 5.

It should be noted that both memory recall mechanisms explained above are not simple replay mechanisms. Rather, both are memory reconstructions with mutations that are caused by spontaneous dynamics. We expect that the memory recall mechanisms will allow Alter3 to explore new movement patterns that cannot be generated from its automatic mimicry capacity. Additionally, the second recall mechanism can select memories in accordance with the ability to imitate humans, for a given memory chunk; therefore, it develops the contents of memory according to the imitation ability. As a result, we expect that memory structures can evolve through the experimental imitations of human agents.

### 3.2. Memory Variation by Spontaneous Dynamics

Alter3 has internal spontaneous dynamics that act as a central pattern generator (CPG). This generator has no input from the environment. It consists of spiking neurons (see Appendix for the details of the neuron model). The first reason for using spiking neurons instead of other chaotic dynamical systems or stochastic dynamic systems is that we intend to add a learning process with stimulus input in the future work (e.g., the difference between simulated future self-image and target human motion might be used as stimulus input to the spiking neurons). The second reason is that, in this research, it is important that memory becomes unstable with the internal dynamics when it is recalled, i.e., the dynamics are used to perturb the memory. Thus, it would be better if the dynamics kept changing with synaptic plasticity. We compared the dynamics of spiking neurons with synaptic plasticity to spiking neurons without synaptic plasticity and random patterns. The results (Figure A1) show that the generated patterns of the spiking neurons with synaptic plasticity were more structured and temporally richer than the ones without plasticity (see Appendix 2 for the details of this analysis). For these reasons, we adopted spiking neurons with plasticity as the candidates for noise sources to perturb memory.

The dynamics of the CPG are added to the motor commands before they are sent to Alter3, which implies that the dynamics also mutate recalled memories, much like memory reconsolidation. The original motor commands generated by automatic imitation or memory selection are always affected by the CPG. Specifically, final motor commands realized by Alter3's hardware are taken in a weighted summation of the original motor commands and output of the CPG. We set the weight of the CPG output to 0.1, and the weight of original motor commands to 0.9. In other words, CPG dynamics mutate recalled memories, like memory reconsolidation.

## 4. Experiments

**Figure 6:**
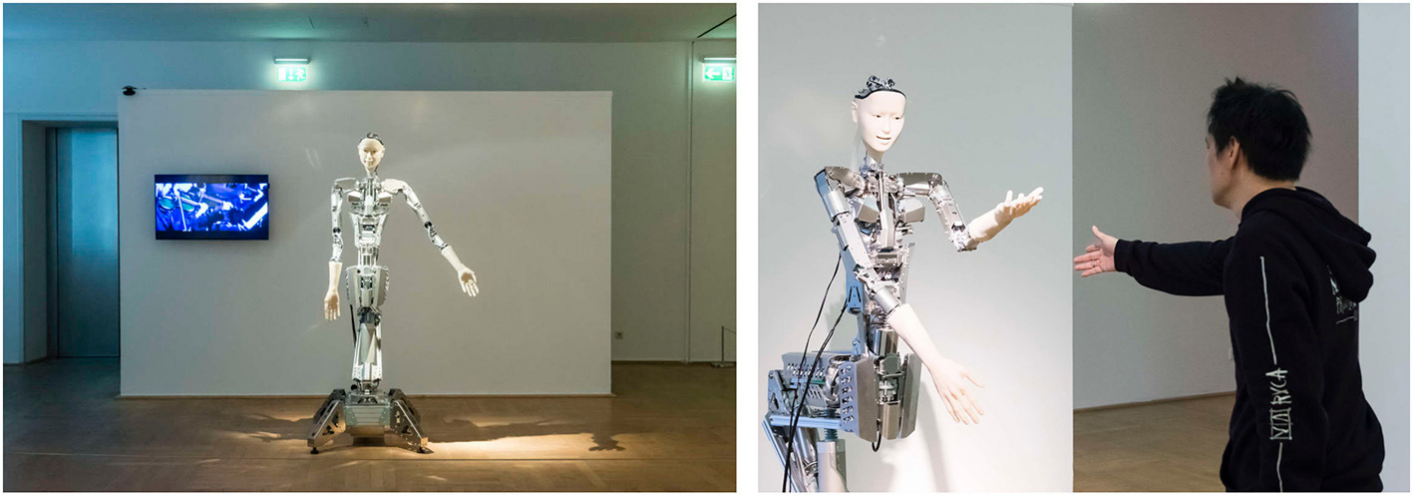
Alter3 at the exhibition NRW-Forum, Düsseldorf. It was evident to the public that Alter3 was trying to imitate the pose of a person.

We conducted experiments with Alter3 at the NRW-Forum, Düsseldorf between April 26 and May 4, 2019. During the experiments, Alter3 was located in the exhibition room (Figure 6, left), which is a public space. The public could freely visit the exhibition and witness Alter3's movements. They were allowed to interact with it through their own movements (Figure 6, right; see also Supplementary Video 1). There was no limitation on the duration for which a person can interact with Alter3, and no information about the experiment was provided besides the fact that Alter3 could imitate human motion. The advantage of a public demonstration was that people of all ages, genders, and nationalities could come to see Alter3. Furthermore, as our policy was to experiment with robots in an open and natural environment, the demonstration was a welcome activity. It is also possible to conduct longer experiments, which can last for weeks (Ikegami, 2010, 2013; Masumori et al., 2020).

**Figure 7:**
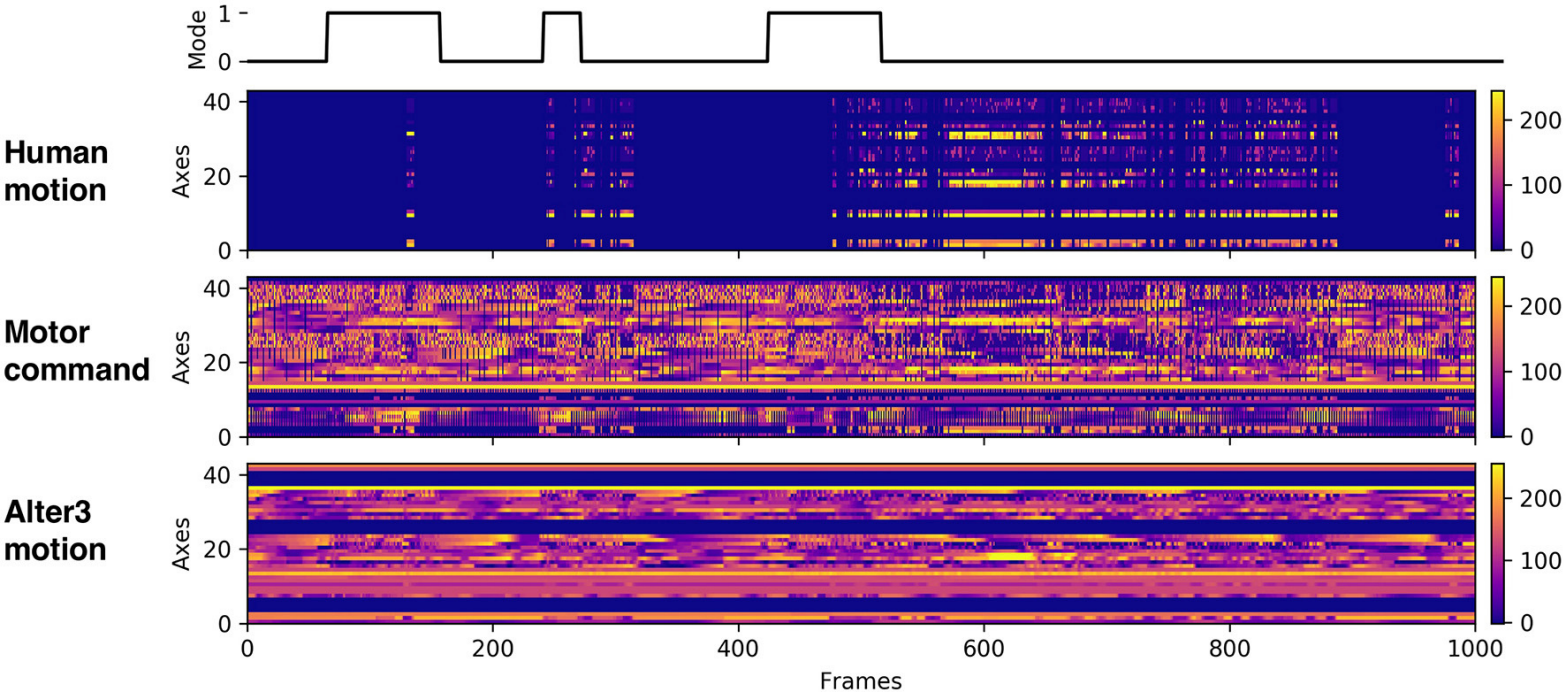
Example of the recorded data. The first raw data represent the mode flag (0 represents awake mode; 1 represents memory mode). The second raw data represent human motion data. The third raw data represent motor command data, which were sent to Alter3. The last raw data represent Alter3's actual motion data.

We performed six experiments, each consisting of 100,000 frames and lasting approximately 4–5 h. During the experiments, we recorded Alter3's motor commands, its actual motion data, and the human motion data (Figure 7). We analyzed these data to understand how Alter3's behavior changed during the experiments.

## 5. Results

### 5.1. Development of Memory Structure

**Figure 8:**
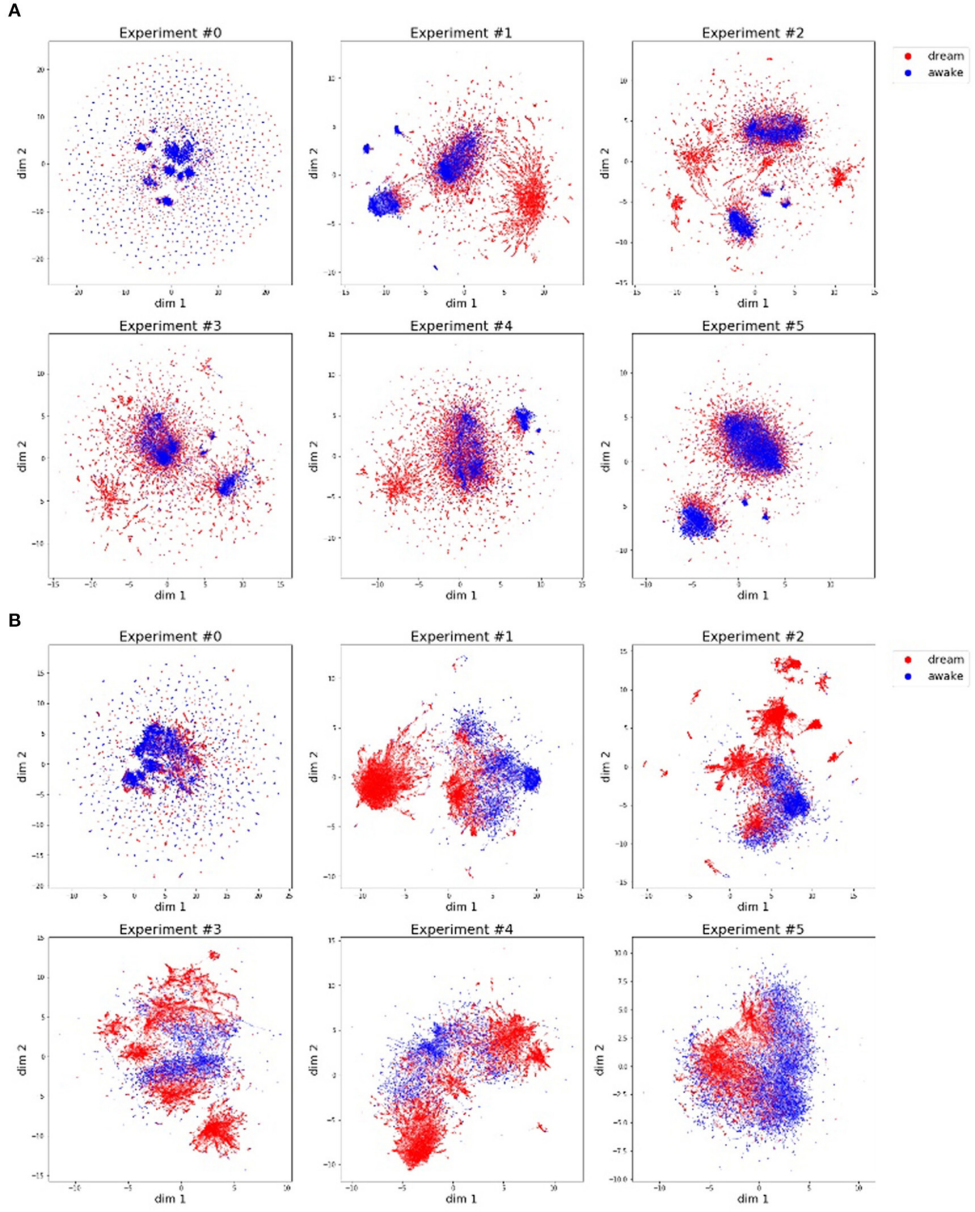
Motor commands **(A)** and motion data **(B)** are projected onto a two-dimensional space using the dimension reducing algorithm, UMAP. The blue dots indicate that the pose is generated by imitating human motion, and the red dots represent poses generated from memory. **(A)** Motor commands data (history of Alter3's motor commands: SETAXIS) for each experiment. **(B)** Motion data (history of Alter3's actual motion: GETAXIS) for each experiment.

We analyzed the change in memory and actual motions of Alter3. The memory and actual motion values (values of SETAXIS and GETAXIS) have 43 dimensions; hence, we adapted a dimension-reduction algorithm called UMAP (McInnes et al., 2018) to visualize them. Figure 8 shows the results of the dimension-reduction by UMAP, which reduced the memory and actual motion data of Alter3 to two dimensions. These results show a different pattern for each experiment, especially experiments #0 and #5. We consider that these differences reflect differences in the interactions between Alter3 and humans. This suggests that different personalities in Alter3 emerge from different environments (e.g., differences in the frequency and duration of people's stays and motion patterns).

As shown in this figure, in almost all the experiments, the poses generated in the awake mode and the poses generated in the dream mode have different clusters, although some of these clusters overlap. The former poses tend to have more clusters than the latter ones. This suggests that Alter3 not only copied human motions but also varied them using its memory mutation and selection process. The memory data (Figure 8A) and the actual motion data (Figure 8B) reflect the same tendencies. However, they also marginally differ because of Alter3's construction: Alter3's axes are controlled by air actuators, and they do not have sufficient torque to control the axes precisely. Thus, the actual motions differ from the motor commands.

**Figure 9:**
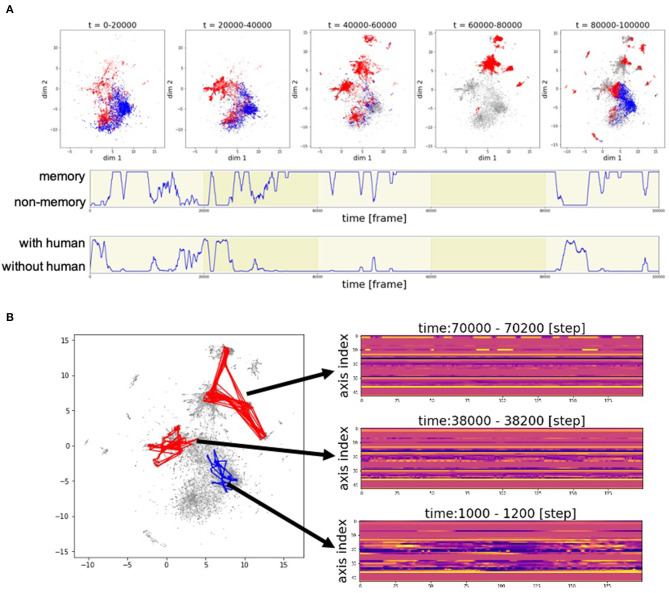
Example of the time development of motion patterns. **(A)** Time series of the poses in two-dimensional space. The entire duration of the experiment was divided into five parts at every 20,000 frames and plotted as five figures (top). The subsequent two rows represent the time series of the switching between memory and awake modes (middle) and the time series of whether a human is within Alter3's sight (bottom). **(B)** Trajectory of motion data in two-dimensional space (UMAP) and sample real data for a point in two-dimensional space. A red line in the two-dimensional space represents the trajectory of the motion data that is generated based on memory. A blue line represents the trajectory of the motion data that is generated based on human imitation.

Figure 9 shows the developments in the motion patterns over time. Figure 9A (top) shows that the clusters of the poses generated from memory, represented by the red dots, are initially located near the clusters of the poses generated by automatic mimicry capacity, represented by the blue dots. Then, the red clusters begin to vary and move away from the blue clusters. At 40,000–60,000 frames and 80,000–100,000 frames, many red clusters can be observed. In these phases, there are cases where Alter3 retrieves a memory and behaves accordingly despite a person being in its sight (Figure 9A, middle and bottom). In such a case, memory selection and the reconsolidation process occur. These results suggest that the memory selection and variation process work well to diversify memory, rather than just copy human motion.

The motion pattern of Alter3 can be represented in a two-dimensional plane. Figure 9B shows the trajectories of the motion data in two-dimensional space (UMAP), and some samples of the data of actual points in the two-dimensional space. It can be observed that the complex motion pattern derived from human motion (at 1000–1200 frames) gradually converges to relatively static motions (at 38,000–38,200 frames and at 70,000–70,200 frames), probably because there were few humans in Alter3's sight at 38,000–38,200 frames, and none at 70,000–70,200 frames. This suggests that Alter3's memory diversifies itself through interactions with the environment (human) at first. However, without such interactions, its memory is overwritten by its spontaneous activity and gradually disappears, similar to forgetting dynamics in actual humans.

### 5.2. Information Flow Between Alter3 and Human

To evaluate whether Alter3 could effectively imitate human motion and whether humans also imitated Alter3, we analyzed the information flow between Alter3 and humans. We used transfer entropy (TE) to estimate the information flow between the motions of Alter3 and the humans during the experiments. TE measures directed information transfer (Schreiber, 2000). A high TE from one entity to another indicates that the former affects the latter. Thus, TE enables us to estimate causation during an imitation.

The TE from time series *J* to time series *I* is defined as

(1) $$\begin{array}{r} TE_{J,I}=\sum p\left(i_{t+1},i_{t}^{\left(k\right)},j_{t+1}^{\left(l\right)}\right)\text{log}\frac{p\left(i_{t+1}|i_{t}^{\left(k\right)},j_{t+1}^{\left(l\right)}\right)}{p\left(i_{t+1}|i_{t}^{\left(k\right)}\right)}, \end{array}$$

where *i*_*t*_ denotes the value of *I* at time *t*, *j*_*t*_ denotes the value of *j* at time *t*, and *i*_*t*+1_ denotes the value of *i* at time *t*+1. Parameters *k* and *l* give the order of the TE and represent the number of time bins in the past that are used to calculate the histories of time series *i* and *j*. Here, they are set to *k* = *l* and *k* = 3.

We computed the TE between the motion data of both Alter3 and humans (continuous multivariate data) using the Kraskov–Stögbauer–Grassberger estimator in the JIDT library (Lizier, 2014) and compared the results for the awake and memory conditions. The awake condition was defined to be equivalent to the awake mode explained above. The memory conditions were defined such that there was a human in front of Alter3, but the error of the optical flow exceeded the threshold, and memory was used to generate Alter3's motion.

**Figure 10:**
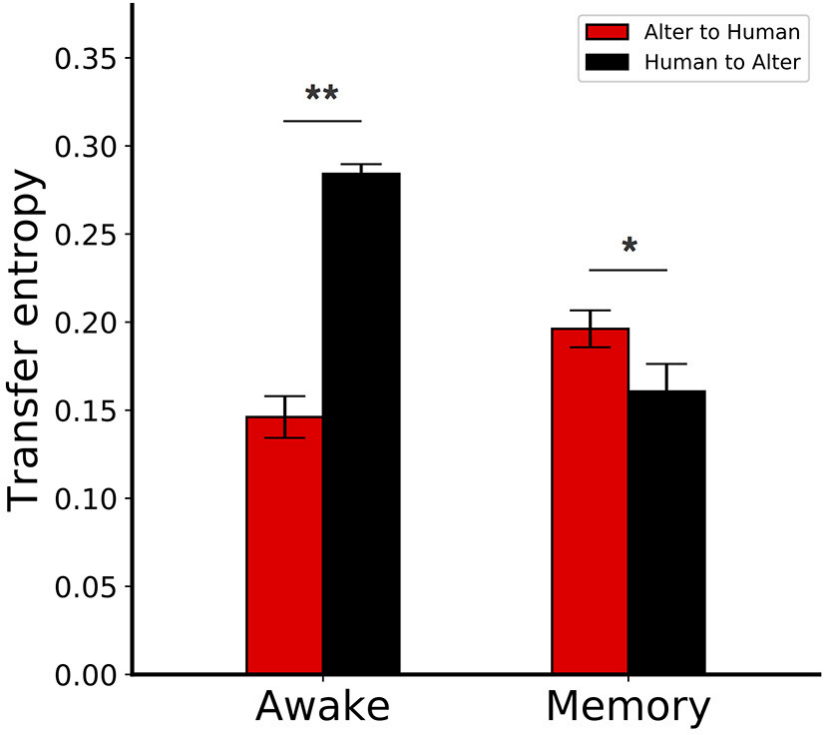
Transfer entropy (TE) between Alter3 and human motions. In the awake condition, the TE from Alter3's motion to human motion was significantly lower than the reverse case. In contrast, for the memory condition, the TE from Alter3 to human motion was significantly higher than the TE in the opposite direction. The awake conditions were equivalent to the awake mode, where Alter3 imitated human motion with its automatic mimicry module. The memory conditions were defined when Alter3 used memory to generate motion (i.e., there was a human in front of Alter3, but the difference between the optical flow values of the human motion image and the simulated future self-image of Alter3 exceeded a threshold; thus, memory was used to generate motion). *p < 0.05, **p < 0.01.

The mean TE values between Alter3's motion and human motion are shown in Figure 10. In the awake mode, the value of TE from Alter3's motion to human motion was significantly lower than in the opposite direction (Mann–Whitney *U*-test, *n* = 6, *p* = 0.0025). This implies that information flow from humans to Alter3 was higher than the flow from Alter3 to humans. This suggests that Alter3 could imitate human motion effectively. In contrast, for the memory condition, the value of TE from Alter3 to human motion was significantly higher than the TE value for the opposite direction (Mann–Whitney *U*-test, *n* = 6, *p* = 0.0227). This suggests that information flow was reversed in the memory condition, and humans tended to imitate Alter3. During the dream mode, the motions were selected from memory based on the similarity of the visual image-based motion pattern (optical flow) between the poses of Alter3 and a human, rather than the similarities of joint angles itself. Thus, under this condition, the similarity of the motion at the joint angle level will not necessarily be as high as it would be in the awake mode. Such a difference may induce people to start imitating Alter3.

**Figure 11:**
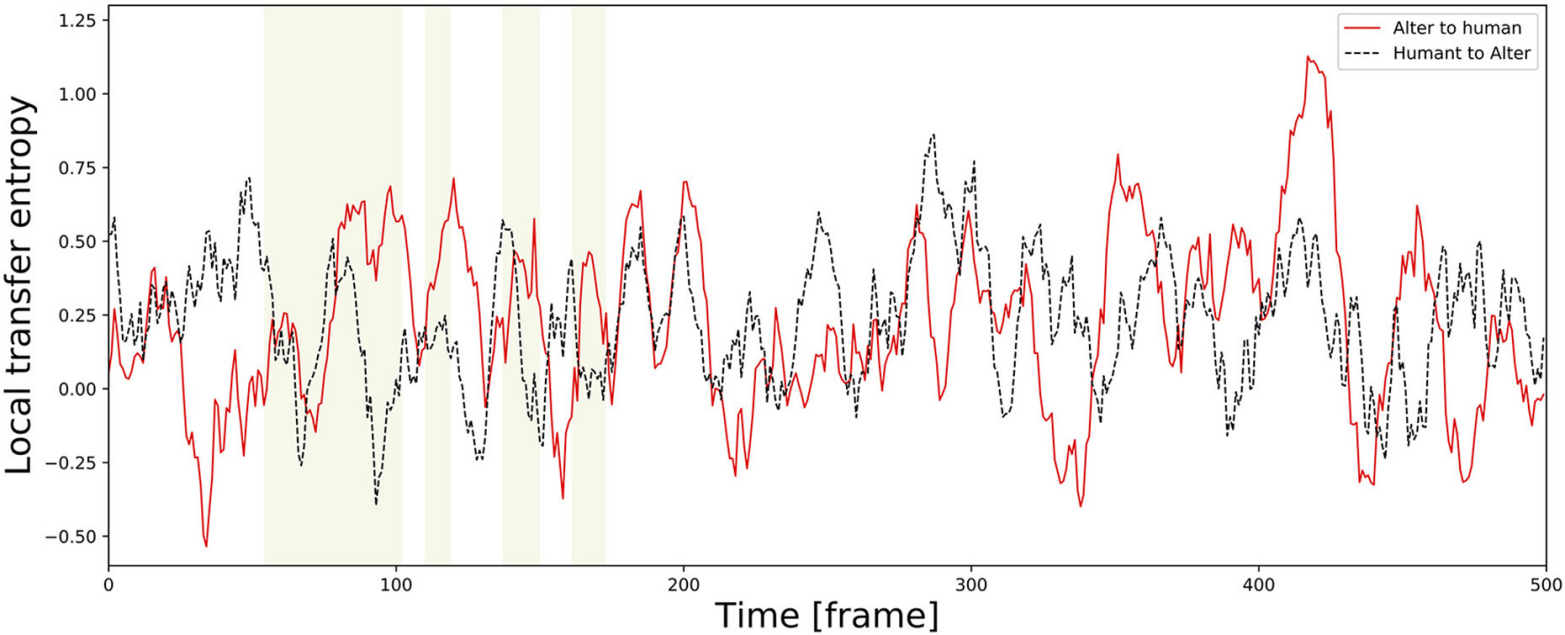
Example of a time series of local transfer entropy (TE) between Alter3 and human motions. The yellow zones indicate a memory condition in the dream mode, where Alter3 used its memory to generate a posture. In the memory condition, the values of local TE from Alter3's motion to human motion tend to be higher than those from human motion to Alter3's motion. Furthermore, in the awake mode, in which Alter3 imitates human motion by automatic mimicry capacity, there were also cases where the values of local TE from Alter3 to human motion were sometimes considerably higher (e.g., close to 420 frames in the figure). We think that this was because Alter3's generated motion pattern was sometimes not as good as expected, thus Alter3 failed to imitate human motion. It seems that this situation led people to imitate Alter3 in turn, thus TE values from Alter3 to human sometimes higher than opposite direction even in the awake mode.

TE varies temporally. As an example of a time series, Figure 11 shows an alternation of local TE between Alter3 and human motions. It shows that, in the memory conditions, the local TE from Alter3's motion to human motion was often higher than in the opposite direction. In addition, in the awake mode, the local TE from Alter3's motion to human motion was sometimes higher than that from human to Alter3. These results imply that the causes and effects of the imitation were often reversed over time; thus, Alter3 and humans imitated each other. We think that this was because Alter3's generated motion pattern was sometimes not as good as expected, thus Alter3 failed to imitate human motion. It seems that such a situation led people to imitate Alter3 in turn, thus the TE value from Alter3 to human was sometimes higher than opposite direction.

## 6. Discussions

Alter3 is programmed to imitate the motion of a person in front of it. A human pose detection algorithm (OpenPose) extracts the key points of the skeleton from the posture pattern. However, Alter3 sometimes fails to imitate the motion. The imitation rating is based on the difference between the optical flow pattern of Alter3 and the optical flow pattern of the person Alter3 attempts to imitate. The smaller the difference, the better the imitation. The main reasons why Alter3 sometimes fails to imitate human motion are (i) physical constraints imposed by the mechanical structure of Alter3, (ii) incorrect detection caused by OpenPose or disturbance to the eye camera, (iii) the dynamic characteristics of Alter3's unstable process, (iv) a significant time delay between the control program and the motor output, and (v) Alter3 encountering a style of motion that cannot be imitated. Such types of failures play an important role for Alter3, such as organizing memory through selection and mutation processes and inducing role switching in interactions with human, as discussed below.

Introducing memory into Alter3, we incorporated an imitation recovery process: if Alter3 fails to imitate human motion with automatic mimicry capacity, Alter3 uses memory to imitate the motion. Alter3's spontaneous neural dynamics commonly affects the generation of motion. Therefore, posture patterns are not only stored in memory but also changed over time. Owing to the selection and the mutation processes, memories are copied and changed when they are used. If Alter3 uses a stored pattern frequently, more copies of this pattern emerge with modifications.

Alter3's organized motion is generated by the automatic mimicry capacity or through Alter3's memory. Therefore, the whole posture space of Alter3 is decomposed into two categories. One consists of the postures provided by estimating human postures, and the other category has the self-organized postures generated through memory selection and variation. The decomposition is shown by applying the UMAP compression in Figure 8. These two categories are created spontaneously through interaction with humans. Moreover, if no person appears in front of Alter3 for a certain period, the postures in the latter category gradually change and converge to Alter3's spontaneous dynamics provided by the spiking neurons, after which another category is organized.

To determine whether Alter3 imitates people's postures or whether people imitate Alter3, we measured the TE between Alter3 and the people whose motions it seemed to imitate. The results suggest that people often imitate Alter3 strongly when Alter3 is in the dream mode (i.e., when Alter3 fails to imitate with the automatic mimicry capacity and it generates a motion from its memory). We also found that people sometimes imitate Alter3, even when Alter3 was in the awake mode (i.e., when it generates a motion based on its autonomous mimicry capacity). It is interesting that people try to imitate the posture of Alter3 because it shows that imitation is an essential property of a living system. In other words, as people grow up, primitive imitation behavior does not disappear, but exists as a background process. For example, close friends are known to synchronize the timing of their speech.

Starting from primitive imitation without any memories, Alter3 develops its memories via imitating human behavior and generates various behaviors based on memory selection and variation processes. While Alter3 interacts with a human and fails to imitate the human's behavior, humans tend to imitate Alter3 instead. This is quantified by the reversal of TE. We say that this spontaneous switching of roles between man and machine is a necessary condition of personogenesis.

## Data Availability Statement

The datasets generated for this study are available on request to the corresponding author.

## Author Contributions

AM and NM contributed equally to this paper, as first authors. All the authors designed the study and the model. AM and NM developed the system, performed the experiment, and analyzed the data. All authors contributed to the interpretation of the results. All authors drafted and revised the article. TI supervised the project.

## Conflict of Interest

AM, NM, and TI are employed by Alternative Machine Inc.
