# Supplementary material for: Personogenesis Through Imitating Human Behavior in a Humanoid Robot “Alter3”
Source: Front Robot AI. 2021 Jan 18;7:532375. doi: 10.3389/frobt.2020.532375 (PMC7849818; doi:10.3389/frobt.2020.532375)
Supplement: Supplementary file 1 [file Data_Sheet_1.pdf]

Appendix

1 SPIKING NEURAL NETWORKS

The model for spiking neurons proposed by Izhikevich (Izhikevich (2003)) was used to simulate excitatory and inhibitory neurons. This model is well known, as it can be regulated to reproduce the dynamics of many variations of the cortical neurons, and it is computationally efficient. The equations of the neural model are defined as:

$$\begin{aligned} &\frac{dv}{dt} = 0.04v^2 + 5v + 140 - u + I, \\ &\frac{du}{dt} = a(bv - u), \\ &\text{if } v \geq 30 \text{ mV}, \text{ then} \begin{cases} v \leftarrow c \\ u \leftarrow u + d \end{cases} \end{aligned} \tag{S1}$$

Here, v represents the membrane potential of the neuron, u represents a variable related to the repolarization of the membrane, I represents noise, t is the time, and a, b, c, and d are other parameters (Izhikevich (2003)) that control the shape of the spike. A neuron is regarded as a firing neuron when its membrane potential $v \geq 30$ mV. The parameters for excitatory neurons (regular-spiking neurons) are set as $a = 0.02,\ b = 0.2,\ c = -65$ mV, and $d = 8$; for inhibitory neurons (fast-spiking neurons), the parameters are set as $a = 0.1,\ b = 0.2,\ c = -65$ mV, and $d = 2$. The simulation time step Δt is 1 ms.

Spike-timing dependent plasticity (STDP) was used as a model for synaptic plasticity, which changes the synaptic weight between two neurons depending on the timing of their spiking (Song et al. (2000)). When the presynaptic neuron fires exactly before the postsynaptic neuron, the synaptic weight increases; when the presynaptic neuron fires exactly after the postsynaptic neuron, the synaptic weight decreases. The weight variation, Δw, is defined as

$$\Delta w = \begin{cases} A_{LTP}(1 - \frac{1}{\tau_{LTP}})^{\Delta t}, & \text{if } \Delta t > 0 \\ -A_{LTD}(1 - \frac{1}{\tau_{LTD}})^{-\Delta t}, & \text{if } \Delta t < 0 \end{cases} \tag{S2}$$

Here, Δt represents the relative spike-timing between a presynaptic neuron a and a postsynaptic neuron b; $\Delta t = t_b - t_a$ (t_a represents the time of the spike of neuron a, and t_b represents the timing of the spike of neuron b). Here, the parameters are set as $A_{LTP}, A_{LTD} = 1.0, \tau_{LTP}, \tau_{LTD} = 20.0$. Δw becomes negative when the postsynaptic neuron fires first and is positive when the presynaptic neuron fires first.

The weight value w between excitatory neurons varies as follows:

$$w_t = w_{t-1} + \Delta w\ . \tag{S3}$$

The maximum possible weight is fixed to $w_{max} = 20.0$ (the values depend on experiments), and if $w > w_{max}$, w is reset to w_{max}. The minimum possible weight is fixed to $w_{min} = 0.0$, and if $w < w_{min}$, w is reset to w_{min}.

We used a network consisting of 1,000 neurons, for the spontaneous dynamics in Alter3. The network consisted of 800 excitatory neurons and 200 inhibitory neurons. The ratio between the excitatory and inhibitory neurons is standard in all the simulations (Izhikevich (2003, 2004)) and is similar to biological values (Cassenaer and Laurent (2007)). The excitatory neurons were divided into two groups: output (10 neurons $\times$ 20 groups) and hidden (600 neurons). The networks were fully connected; the weight values w between each neuron were randomly initialized with uniform distributions in the range of $0 < w < 5$ for excitatory neurons and $-5 < w < 0$ for inhibitory neurons. Only connections between excitatory neurons had synaptic plasticity based on STDP, and the weight values of other connections did not change.

The refresh rate of the simulation was 60 Hz. For sending motion commands in Alter3, at every step, the number of spiked neurons in each output group was weighted and summed up with each original motor command, as explained above.

2 DYNAMICS OF SPIKING NEURONS WITH SYNAPTIC PLASTICITY

We simulated spiking neural networks consisting of 1,000 neurons with and without STDP, for 500,000 steps. The parameters used in the simulations were the same as the parameters used in the main experiments. We sampled one output group consisting of 10 neurons, and the time series of the spiked neurons in the group were used as a generated pattern, for 30 steps (i.e., [0—10] $\times$ 30 steps). We also prepared random patterns consisting of uniform random numbers, where the dimensions were the same as the generated patterns by spiking neurons. As shown in Fig. S1, we visualized the generated patterns using UMAP, which reduced the dimensions of the data. The results qualitatively show that the generated patterns of the spiking neurons with synaptic plasticity (Fig. S1A) were more structured and temporally richer than the patterns obtained from spiking neurons without plasticity and random noise(Fig. S1B,C). In the first temporal stages, there is no apparent difference between plastic and non-plastic networks; however, the difference gradually increases in the latter stages, and cluster-like structures are created for the case of plastic neurons (red dots in the figure).

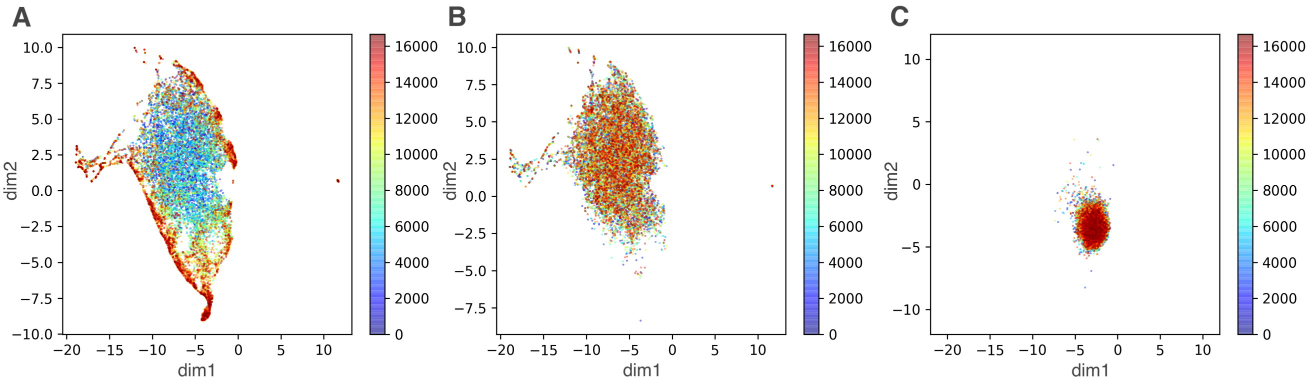

Figure S1. Patterns generated by spiking neurons with/without spike-timing dependent plasticity (STDP), and random numbers reduced to two dimensions using the UMAP algorithm. Each dot represents a generated pattern. Color represents time steps. **A**. Spiking neurons with STDP. **B**. Spiking neurons without STDP. **C**. Random patterns.

REFERENCES

Cassenaer, S. and Laurent, G. (2007). Hebbian STDP in mushroom bodies facilitates the synchronous flow of olfactory information in locusts. *Nature* 448, 709–713. doi:10.1038/nature05973

Izhikevich, E. M. (2003). Simple model of spiking neurons. *IEEE Transactions on Neural Networks* 14, 1569–1572. doi:10.1109/TNN.2003.820440

Izhikevich, E. M. (2003). Simple model of spiking neurons. *IEEE Transactions on Neural Networks* 14, 1569–1572. doi:10.1109/TNN.2003.820440

Izhikevich, E. M. (2004). Which model to use for cortical spiking neurons? *IEEE Transactions on Neural Networks* 15, 1063–1070. doi:10.1109/TNN.2004.832719

Song, S., Miller, K. D., and Abbott, L. F. (2000). Competitive Hebbian learning through spike-timing-dependent synaptic plasticity. *Nature neuroscience* 3, 919–926. doi:10.1038/78829
